# Design, Synthesis and Pharmacological Evaluation of Naphthofuran Derivatives as Potent SIRT1 Activators

**DOI:** 10.3389/fphar.2021.653233

**Published:** 2021-04-28

**Authors:** Jian Gao, Qing-Qing Chen, Ye Huang, Kai-Hang Li, Xiao-Ju Geng, Tao Wang, Qi-Si Lin, Ruo-Si Yao

**Affiliations:** ^1^ Jiangsu Key Laboratory of New Drug Research and Clinical Pharmacy, Xuzhou Medical University, Xuzhou, China; ^2^ Xuzhou Medical University Technology Transfer Center Co., Ltd., Xuzhou Medical University, Xuzhou, China; ^3^ Jiangsu College of Nursing, Huaian, China; ^4^ Department of Hematology, The Affliated Hospital of Xuzhou Medical University, Xuzhou, China; ^5^ Blood Diseases Institute, Xuzhou Medical University, Xuzhou, China

**Keywords:** diabetic nephropathy, SIRT1 activators, virtual screening, apoptosis, inflammation

## Abstract

Diabetic nephropathy (DN) is one of the most important medical complications in diabetic patients, which is an essential cause of end-stage renal disease in diabetic patients and still lacks effective medicines. Silent information regulator 1 (SIRT1) is closely related to the occurrence and development of DN. Activation of SIRT1 could significantly improve the symptoms of DN, while the activities of SIRT1 activators need to be further improved. Based on the crystal structure of SIRT1, structure and ligand-based approaches were carried out, and a lead compound 4,456–0661 (renamed as M1) was identified. Moreover, seven M1 analogues (6a-6g) were designed using a structure-based drug design strategy followed by bioactivity evaluation with SRTR2104 used as positive drugs. Among the target molecules, compounds M1, 6b, and 6d were proved to be potent SIRT1 activators, the activities of which are comparable to SRT2104. More importantly, compounds M1, 6b, and 6d could resist high glucose-induced apoptosis of HK-2 cells by activating SIRT1 and deacetylation of p53. Apart from the beneficial effect on apoptosis of DN, these compounds also alleviated high glucose stimulating inflammation response in HK-2 cells through SIRT1/NF-κB (p65) pathway. Consequently, M1, 6b, and 6d could be promising drug candidates for SIRT1 related diseases.

## Introduction

As per international diabetes federation data, 463 million adults were living with diabetes worldwide in 2019, and this number would double by the year 2045. Among them, the current number of diabetic patients in China is as high as 114 million, still ranking first in the world (Saeedi et al., 2019). Diabetic nephropathy (DN) is one of the most critical medical complications in diabetic patients. It is one of the most important factors that cause end-stage renal disease (ESRD) in diabetic patients. Its higher morbidity and mortality have significantly increased the economic burden on patients with diabetes and society (Hsu et al., 2009; Flyvbjerg, 2017). However, to date, there is still a lack of effective drugs and approaches to halt the progression of DN.

**FIGURE 1:**
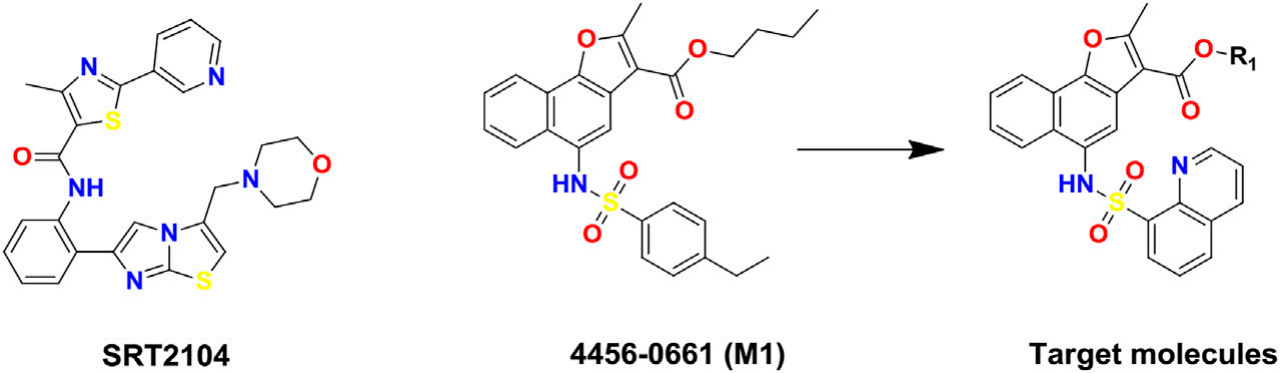
Chemical structures of SIRT1 activators: SRT2104, lead compound 4,456–0661 (M1) and its naphthofuran derivatives.

Accumulating evidence has demonstrated that p53 plays a vital role in the genesis and development of DN (Deshpande et al., 2013; Saito et al., 2016; Guo et al., 2018). Studies have shown that p53 was elevated in kidney cortex of diabetic rodents (Tikoo et al., 2008; Deshpande et al., 2013; Guo et al., 2018) and is elevated in renal biopsies of DN patients (Saito et al., 2016), while acetylation of p53 is essential for its stabilization and function (Brooks et al., 2003). Acetylation could stabilize and activate p53, thereby promoting the transcription of target genes (Ong and Ramasamy, 2018). In addition, p53 is also one of the crucial substrates of silent information regulator 1 (SIRT1) (Vaziri et al., 2001; Cheng et al., 2003; Kim et al., 2007), and SIRT1 activators might protect against DN through the deacetylation of p53 (Ma et al., 2019). In addition, inflammation is a major mechanism in the pathogenesis of DN (Wada and Makino, 2013). Down-regulation of SIRT1 caused by diabetes leads to the activation of NF-κB signaling, thereby promoting the activation of downstream inflammatory factors (Chen et al., 2001; Greene and Chen, 2004). The typical pathway of NF-κB activation involves the p65 and p50 subunits (Gasparini and Feldmann, 2012). SIRT1 is now known to deacetylate the p65 subunit of NF-κB and inhibit the pro-inflammatory signals of NF-κB (Gasparini and Feldmann, 2012; Guo et al., 2014; Du et al., 2018). Therefore, SIRT1 can reduce inflammation in DN through the deacetylation of NF-κB (Li et al., 2017). SRT2104 (Figure 1) is a novel, highly selective small-molecule SIRT1 activator (Hoffmann et al., 2013), which can improve DM-induced aortic endothelial dysfunction in mice and have a protective effect on DN (Brooks et al., 2003; Wu et al., 2018). Therefore, there is an urgent need to develop more and more SIRT1 activators with novel scaffolds.

In this study, to discover novel and potent SIRT1 activators, structure and ligand-based virtual screening (VS) toward the N-terminal domain (NTD) of SIRT1 was conducted, and 37 compounds were selected for SIRT1 assay activity. Among the identified hits, compound 4,456–0661 (simplified as M1, Figure 1) exhibited the best SIRT1 activation effect. Furthermore, based on compound M1, we firstly synthesized a series of novel naphthofuran derivatives (Figure 1) and then evaluated their experimental bioactivity testing. Also, the binding modes of the active compounds to SIRT1 were also studied in this context.

## Materials and Methods

### Molecular Docking-Based Virtual Screening

The crystal structure of the SIRT1 complex activator [PDB ID: 4ZZH (Dai et al., 2015)] was used for molecular docking-based virtual screening, and ChemDiv database (commercially available and the purity of compounds are greater than 95%) of TopScience Co., Ltd. (Shanghai, China) was selected as a screening library. In view of only 2D structural information available, the compounds in the ChemDiv database were preprocessed by the dbtranslate module in Sybyl-X2.1 (Sybyl-X2.1 is available from Tripos Associates Inc., S Hanley Rd., St. Louis, MO 631444, United States). Moreover, Lipinski’s rule of five (Ro5) (Lipinski et al., 2001) was firstly employed on the ChemDiv database to eliminate non-drug-like molecules.

The binding pocket formed by Thr209, Ile210, Pro211, Pro212, Leu215, Thr219, Gln222, Asn226, and Glu230 on the NTD of SIRT1 was regarded as the potential binding site of SIRT1 activators as described in previous study (Dai et al., 2015). The binding site is located in the N-terminal domain of SIRT1, which aims for the specific allosteric activation mechanism of SIRT1 activator. During protein preparation, the missing hydrogen atoms of SIRT1 were added by biopolymer module and all water molecules were removed. To accelerate the virtual screening, a high-speed screening was carried out by decreasing the maximum quantity of conformations and rotatable bonds from 20 to 10, and from 100 to 50, respectively. Then, the molecules with docking score within the top 1% were normative screened again using the default docking parameters, which would bring out the top 500 molecules. Finally, several compounds were selected for commercially purchase by ranking the docking scores and clustering analysis, and followed by *in vitro* SIRT1 enzymatic assay.

### General Synthetic Procedures of the Target Compounds 6a-g

4-amino-1-naphthol (1.96 g, 10 mmol) was dissolved in pyridine (50 ml). After cooling in an ice bath (0°C), the dichloromethane solution (20 ml) of quinoline-8-sulfonyl chloride (2.28 g, 10 mmol) was added dropwise, and then stirred at room temperature for 4 h. After removing the solvent *in vacuo*, the residue was dissolved in EtOAc (50 ml), which was then washed with 1N HCl, distilled water, and brine. The organic layer was dried over anhydrous MgSO_4,_ filtered, and evaporated *in vacuo*. The residue was recrystallized from absolute ethanol to obtain compound 1.

K_2_Cr_2_O_7_ (664.9 mg) was added in acetic acid (16.6 ml) and stirred at 20°C for 1 h. Then, compound 1 (990 mg) was added and continued to stir at 20°C for another 2.5 h. After the reaction was complete, the solution was poured into cold water to obtain yellow precipitate 2.

The crude intermediate 2 (1.2 g) and ethyl acetoacetate (480 μL) was dissolved in 1, 4-dioxane (5 ml) and stirred at 25°C for 5 min, then sodium methoxide (15 mg) was added. After stirring at 25°C for another 30 min, the solution was evaporated *in vacuo* to give a residue, which was recrystallized from absolute ethanol to obtain compound 3.

Compound 3 (576.4 mg) was dissolved in acetic acid (2.11 ml), and then sulfuric acid (158.9 μL) was added in the solution slowly. After stirring at 118°C for 30 min, the solution was poured into ice water, and neutralized by Na_2_CO_3_ to give the crude precipitate, which was further purified by column chromatography to obtain compound 4.

Compound 4 (972 mg) was dissolved in methanol, then 15% K_2_CO_3_ (14 ml) solution was added. After refluxing for 12 h, the solution was concentrated *in vacuo* to remove methanol. The residue was acidified using 2N HCl (final pH = 3). Precipitate was filtered, and recrystallized from methanol to obtain compound 5.

Compound 5 (100 mg) was dissolved in various alcohols, and concentrated sulfuric acid (26.23 μL) was added to the solution slowly. After stirring at 100°C for 9 h, the alcohol was removed *in vacuo*. Ice water was then added to the residue, and neutralized by 10% NaOH (final pH = 6.5). Precipitate was filtered, and recrystallized from EtOAc to give the target compounds 6a-g, respectively.

#### Methyl 2-Methyl-5-(Quinoline-8-Sulfonamido)Naphtho[1,2-b] Furan-3-Carboxylate (6a)

As a white powder, yield 62.4%, m. p.191.8–192.9°C; Analytical data for ***6a***: ^1^H NMR (400 MHz, CDCl_3_, *δ* ppm): 9.26 (d, *J* = 4.0 Hz, 1H, Ar-H), 8.60 (s, 1H, NH), 8.50 (d, *J* = 8.4 Hz, 1H, Ar-H), 8.40 (d, *J* = 8.0 Hz, 1H, Ar-H), 8.28 (d, *J* = 7.2 Hz, 1H, Ar-H), 8.17 (d, *J* = 8.0 Hz, 1H, Ar-H), 8.10 (d, *J* = 8.4 Hz, 1H, Ar-H), 7.68 (dd, *J* = 8.4, 4.4 Hz, 1H, Ar-H), 7.70–7.50 (m, 3H, Ar-H), 7.15 (s, 1H, Ar-H), 3.58 (s, 3H, OCH_3_), 2.74 (s, 3H, CH_3_); ^13^C NMR (100 MHz, DMSO, δ ppm): 164.48, 163.25, 151.76, 148.21, 143.62, 137.44, 136.09, 133.55, 131.84, 129.34, 129.17, 128.09, 127.29, 126.15, 125.99, 125.07, 122.99, 121.26, 120.85, 119.90, 117.07, 109.93, 51.15, 14.52; ESI-MS: m/z 447.1 [M + H]^+^.

#### Ethyl 2-Methyl-5-(Quinoline-8-Sulfonamido)Naphtho[1,2-b]Furan-3-Carboxylate (6b)

As a yellow powder, yield 82.0%, m. p.193.2–194.1°C; Analytical data for 6b: ^1^H-NMR (400 MHz, CDCl_3_, δ ppm): 9.25 (dd, *J* = 4.4, 1.6 Hz, 1H, Ar-H), 8.68 (s, 1H, NH), 8.52 (d, *J* = 8.4 Hz, 1H, Ar-H), 8.39 (dd, *J* = 8.4, 1.6 Hz, 1H, Ar-H), 8.29 (dd, *J* = 7.2, 1.2 Hz, 1H, Ar-H), 8.20 (d, *J* = 8.0 Hz, 1H, Ar-H), 8.10 (dd, *J* = 8.4, 1.6 Hz, 1H, Ar-H), 7.70 (dd, *J* = 8.4, 4.4 Hz, 1H, Ar-H), 7.62–7.52 (m, 3H, Ar-H), 7.27 (d, *J* = 4.8 Hz, 1H, Ar-H), 4.08 (q, *J* = 7.2 Hz, 2H, CH_2_), 2.77 (s, 3H, CH_3_), 1.04 (t, *J* = 6.8 Hz, 3H, CH_3_); ^13^C NMR (100 MHz, CDCl_3_, δ ppm): 164.15, 163.21, 151.70, 148.23, 143.63, 137.45, 136.23, 133.54, 131.67, 129.37, 129.15, 127.30, 126.16, 125.98, 125.11, 122.61, 121.31, 121.00, 119.94, 116.91, 110.15, 60.14, 14.57, 14.44; ESI-MS: m/z 461.1 [M + H]^+^.

#### 2-Hydroxyethyl 2-Methyl-5-(Quinoline-8-Sulfonamido)Naphtha[1,2-b]Furan-3-Carboxylate (6c)

As a yellow powder, yield 38.1%, m. p.196.5–197.8°C; Analytical data for ***6c***: ^1^H-NMR (400 MHz, DMSO, δ ppm): 9.99 (s, 1H, NH), 9.22 (dd, *J* = 4.0, 1.6 Hz, 1H, Ar-H), 8.59 (dd, *J* = 8.4, 1.6 Hz, 1H, Ar-H), 8.29–8.25 (m, 2H, Ar-H), 8.15 (dd, *J* = 7.6, 1.6 Hz, 1H, Ar-H), 8.10 (d, *J* = 8.4 Hz, 1H, Ar-H), 7.81 (dd, *J* = 8.4, 4.4 Hz, 1H, Ar-H), 7.63–7.60 (m, 2H, Ar-H), 7.45 (s, 1H, Ar-H), 7.40 (t, *J* = 7.2 Hz, 1H, Ar-H), 4.13 (t, *J* = 5.2 Hz, 2H, CH_2_), 3.50 (t, *J* = 5.2 Hz, 2H, CH_2_), 2.75 (s, 3H, CH_3_); ^13^C NMR (100 MHz, DMSO, δ ppm): 163.00, 162.91, 151.51, 146.67, 142.96, 137.11, 135.87, 133.98, 131.38, 129.61, 128.55, 128.18, 127.21, 125.64, 125.28, 124.97, 122.66, 120.65, 120.03, 119.14, 117.27, 109.31, 65.46, 58.94, 14.25; ESI-MS: m/z 477.1 [M + H]^+^.

#### Propyl 2-Methyl-5-(Quinoline-8-Sulfonamido)Naphtho[1,2-b]Furan-3-Carboxylate (6d)

As a white powder, yield 63.8%, m. p.237.8–238.6°C; Analytical data for 6d:1H-NMR (400 MHz, CDCl_3_, δ ppm): 9.26 (s, 1H, Ar-H), 8.78 (s, 1H, NH), 8.49 (d, *J* = 8.4 Hz, 1H, Ar-H), 8.41 (d, *J* = 6.4 Hz, 1H, Ar-H), 8.29 (d, *J* = 7.2 Hz, 1H, Ar-H), 8.19 (d, *J* = 8.0 Hz, 1H, Ar-H), 8.09 (d, *J* = 8.0 Hz, 1H, Ar-H), 7.71 (s, 1H, Ar-H), 7.59 (t, *J* = 7.6 Hz, 2H, Ar-H), 7.52 (t, *J* = 7.2 Hz, 1H, Ar-H), 7.34 (s, 1H, Ar-H), 4.02 (t, *J* = 6.8 Hz, 2H, CH_2_), 2.77 (s, 3H, CH_3_), 1.45–1.40 (m, 2H, CH_2_), 0.87 (t, *J* = 7.2 Hz, 3H, CH_3_); ^13^C NMR (100 MHz, CDCl_3,_ δ ppm): 164.23, 163.13, 151.61, 148.21, 143.49, 137.80, 136.17, 133.55, 131.78, 129.31, 129.18, 129.06, 127.27, 126.24, 125.98, 125.01, 122.63, 121.28, 121.09, 119.95, 116.97, 110.21, 65.86, 22.15, 14.62, 10.63. ESI-MS: m/z 475.2 [M + H]^+^.

#### 3-Hydroxypropyl 2-Methyl-5-(Quinoline-8-Sulfonamido)Naphtho[1,2-b]Furan-3-Carboxylate (6e)

As a white powder, yield 57.3%, m. p.188.9–190.3°C; Analytical data for **6e**: ^1^H-NMR (400 MHz, CDCl_3,_ *δ* ppm): 9.25 (dd, *J* = 4.4, 1.6 Hz, 1H, Ar-H), 8.70 (s, 1H, NH), 8.37–8.35 (m, 2H, Ar-H), 8.30 (dd, *J* = 7.2, 1.2 Hz, 1H, Ar-H), 8.16 (dd, *J* = 7.3, 1.4 Hz, 1H), 8.07 (dd, *J* = 8.4, 1.2 Hz, 1H, Ar-H), 7.69 (dd, *J* = 8.4, 4.4 Hz, 1H, Ar-H), 7.58–7.54 (m, 2H, Ar-H), 7.50–7.44 (m, 2H, Ar-H), 5.30 (s, 1H, OH), 4.29 (t, *J* = 6.0 Hz, 2H, CH_2_), 3.70 (t, *J* = 6.0 Hz, 2H, CH_2_), 2.76 (s, 3H, CH_3_), 1.83–1.77 (m, 2H, CH_2_); ^13^C NMR (100 MHz, CDCl_3,_ *δ* ppm): 164.34, 163.06, 151.38, 147.65, 143.22, 137.25, 135.80, 133.39, 131.40, 129.02, 128.81, 128.01, 126.89, 125.75, 125.58, 124.09, 122.33, 120.89, 120.75, 119.72, 116.03, 109.69, 60.75, 58.76, 31.72, 14.31; ESI-MS: m/z 491.1 [M + H]^+^.

#### Butyl 2-Methyl-5-(Quinoline-8-Sulfonamido)Naphtho[1,2-b]Furan-3-Carboxylate (6f)

As a white powder, yield 38.9%, m. p.210.8–211.2°C; Analytical data for ***6f***: ^1^H-NMR (400 MHz, CDCl_3_, *δ* ppm): 9.24 (s, 1H, Ar-H), 8.74 (s, 1H, NH), 8.47 (d, *J* = 8.4 Hz, 1H, Ar-H), 8.40 (d, *J* = 8.0 Hz, 1H, Ar-H), 8.29 (d, *J* = 6.8 Hz, 1H, Ar-H), 8.18 (d, *J* = 8.0 Hz, 1H, Ar-H), 8.08 (d, *J* = 8.0 Hz, 1H, Ar-H), 7.70 (dd, *J* = 8.0, 4.0 Hz, 1H, Ar-H), 7.58 (t, *J* = 7.2 Hz, 2H, Ar-H), 7.51 (t, *J* = 7.6 Hz, 1H, Ar-H),7.36 (s, 1H, Ar-H), 4.08 (t, *J* = 6.8 Hz, 2H, CH_2_), 2.77 (s, 3H, CH_3_), 1.49–1.29 (m, 4H, CH_2_CH_2_), 0.95 (t, *J* = 7.2 Hz, 3H, CH_3_); ^13^C NMR (100 MHz, CDCl_3,_ *δ* ppm): 164.23, 163.05, 151.64, 148.14, 143.38, 137.69, 136.20, 133.57, 131.70, 129.30, 129.13, 128.91, 127.23, 126.16, 125.94, 124.90, 122.63, 121.25, 121.14, 119.95, 116.88, 110.22, 64.12, 30.80, 19.34, 14.64, 13.96. ESI-MS: m/z 489.2 [M + H]^+^.

#### 4-Hydroxybutyl 2-Methyl-5-(Quinoline-8-Sulfonamido)Naphtho[1,2-b]Furan-3-Carboxylate (6g)

As a yellow powder, yield 38.9%, m. p.191.2–192.3°C; Analytical data for ***6g***: ^1^H-NMR (400 MHz, CDCl_3_, *δ* ppm): 9.25 (d, *J* = 2.8 Hz, 1H, Ar-H), 8.72 (s, 1H, NH), 8.37 (dd, *J* = 8.4, 1.2 Hz, 1H, Ar-H), 8.30–8.26 (m, 2H, Ar-H), 8.17 (d, *J* = 8.0 Hz, 1H, Ar-H), 8.07 (dd, *J* = 8.4, 1.2 Hz, 1H, Ar-H), 7.69 (d, *J* = 8.4, 4.0 Hz, 1H, Ar-H), 7.59–7.52 (m, 3H, Ar-H), 7.42 (t, *J* = 8.5 Hz, 1H), 4.20 (t, *J* = 6.0 Hz, 2H, CH_2_), 3.74 (t, *J* = 5.6 Hz, 2H, CH_2_), 2.78 (s, 3H, CH_3_), 1.73–1.70 (m, 4H, CH_2_CH_2_); ^13^C NMR (100 MHz, CDCl_3_, *δ* ppm): 164.34, 163.31, 151.68, 147.96, 137.54, 136.30, 133.67, 131.55, 129.18, 129.14, 128.20, 127.73, 126.09, 125.84, 124.22, 122.65, 121.21, 121.18, 120.11, 116.79, 110.24, 64.28, 62.60, 29.99, 25.49, 14.63. ESI-MS: m/z 505.2 [M + H]^+^.

### Biological Assays

Human renal tubular epithelial cell line (HK-2) and human mesangial glomerular cell (HMC) were kindly provided by The Xuzhou Medical University, Xuzhou, China. SRT2104 was purchased from Selleckchem and CCK-8 kit was purchased from VICMED.

### Cell Culture

HK-2 and HMC cell lines was cultured in DMEM/low glucose (5.5 mM) containing 10% fetal bovine serum (FBS). The cell line was grown in a humidified incubator at 37°C in the presence of 5% CO_2_.

### *In Vivo* Cytotoxicity Assay

CCK-8 assays were performed to determine SIRT1 agonists cytotoxicity. Briefly, 5,000 HK-2 cells in logarithmic phase were seeded in a 96-well plate and kept at 37°C and 5% CO_2_ overnight. Then cells were treated with different concentrations of compounds (5, 10, 20, 40, 80, and 100 μM dissolved in DMSO) for 48 h. Following incubation, cells were treated with 5 μl CCK-8 solution for 3 h, then the cytotoxicity was determined by a spectrophotometer set (BioTek, Winooski, VT, United States) at a wavelength of 450 nm. SRT2104 was used as positive control.

### *In Vitro* SIRT1 Assay

SIRT1 activation assay for the target molecules was performed using SIRT1 fluorometric drug discovery kit (GMS50289.1 v. A, GENMED SCIENTIFICS INC. United States). As per the supplier protocol, all samples must be processed by adding GENMED lysis solution (Reagent A), GENMED separation solution (Reagent B), GENMED cleaning solution (Reagent C), and GENMED extraction solution (Reagent D). Placed the prepared samples in an ice bath, added corresponding amounts of GENMED buffer (Reagent E), GENMED substrate solution (Reagent F), GENMED supplement (Reagent I), GENMED stop solution (Reagent G) and GENMED enzymatic hydrolysis solution (Reagent H), then gently shaked gently for 30 s, react at 30°C for 30 min, and immediately put it into the fluorescence microplate reader (excitation set at 355 nm and emission measured at 460 nm) so as to obtain the relative fluorescence unit (RFU) reading. Finally, the sample's fluorescence reading RFU was regarded as the sample enzyme activity unit, set the enzyme activity of the blank group to 1, and obtained the different samples relative enzyme activity. The experiment was repeated three times. The detection is based on the synthetic acetylated p53 (residues from 379 to 382) polypeptide substrate Ac-Arg-His-Lys-Lys (Ac), which has the function of anti-aminopeptidase cleavage. First, use the fluorescent dye aminomethyl coumarone to label the acetylated p53 polypeptide substrate, followed by deacetylation, and finally the substrate is further catalyzed by aminopeptidase to release 7-amino-4 methylcoumarin with strong fluorescence. To assess the specificity of the target molecules among mammalian sirtuins, SIRT2 (GMS50786.1), and SIRT3 (GMS50288.1) activation assays were carried out using the similar fluorometric drug discovery kit.

### Apoptosis Analysis

For apoptotic detection, indicated cells treated with different doses of SIRT1 agonists were suspended with 100 μL binding buffer, and incubated with fluorescein isothiocyanate (FITC)-conjugated Annexin V (KeyGEN, China). The rate of apoptosis was detected by flow cytometry as described in previous reports (Yao et al., 2018; Yao et al., 2020).

### Western Blotting

Indicated HK-2 cells treated with SIRT1 agonists were lyzed in RIPA lysis buffer with 1 mM phenylmethylsulfonyl fluoride (PMSF). Whole protein extracts were electrophoresed on SDS polyacrylamide gels and transferred to polyvinylidene fluoride (PVDF) membranes followed by blocking with 5% non-fat dry milk in Tris buffered saline and 0.1% Tween (TBST) at room temperature. The membranes were incubated overnight at 4°C with primary antibodies diluted in blocking solution (anti-SIRT1: 1:1,000, NF-κB (p65) and p53: 1:1,000, Acetyl-p65 and p53: 1:500 and anti-β-actin: 1:2,000). After washing in TBST, the corresponding horseradish peroxidase-conjugated secondary antibodies (horseradish peroxidase-conjugated rabbit IgG and horseradish peroxidase-conjugated mouse IgG: 1:5,000) were added for 1 h. Protein bands were visualized by chemiluminescence using Western ECL substrate (Bio-Rad, Hercules, CA, United States). The densitometric analysis of the bands was conducted by ImageJ 1.46 software (National Institutes of Health, Bethesda, MD, United States).

### Q-PCR Assay

Total RNA was extracted using TRIzol total RNA isolation reagent (Gibco, Thermo Fisher Scientific, Inc.). cDNA was synthesized using the RT-PCR kit. Primer sequences were as follows: p53, forward 5′-GGC​CAT​CTA​CAA​GCA​GTC​A-3′ and reverse 5′-GGG​CAG​TGC​TCG​CTT​AG-3′; MCP-1, forward 5′-TGT​CTG​GAC​CCA​TTC​CTT​CT-3′ and reverse 5′-ACC​AGC​AAG​ATG​ATC​CCA​AT-3′; ICAM-1, forward 5′-CGACTGGACGAGAGG GATTG-3′ and reverse 5′-TTA​TGA​CTG​CGG​CTG​CTA​CC-3′; and β-actin, forward 5′-TCG​TGC​GTG​ACA​TTA​AGG​AG-3′ and reverse 5′-ATG​CCA​GGG​TAC​ATG​GTG​GT-3′. The Q-PCR system contained: Sense primer 0.5 μl (10 μM), antisense primer 0.5 μl (10 μM), cDNA (1 μl), double distilled H_2_O (8 μl) and SYBR Supermix from the iTaq™ Universal Syber green One Step kit (10 μl). Expression levels of mRNA were quantified by the 2^−ΔΔCt^ method.

## Results and Discussion

### Chemistry

All of the synthesized compounds were purified by column chromatography on silica gel 60 (Qingdao Ocean Chemical Company, China). Melting points of individual compounds were determined on a model YRT-3 apparatus and uncorrected. ^1^H NMR (400 MHz) and ^13^C NMR (100 MHz) spectra were performed on a JEOL (400 MHz). MS detection was performed using an Agilent G6125BA single quadrupole mass spectrometer. All solvents were reagent grade and, when necessary, were purified and dried by standard methods.

**SCHEME 1:**
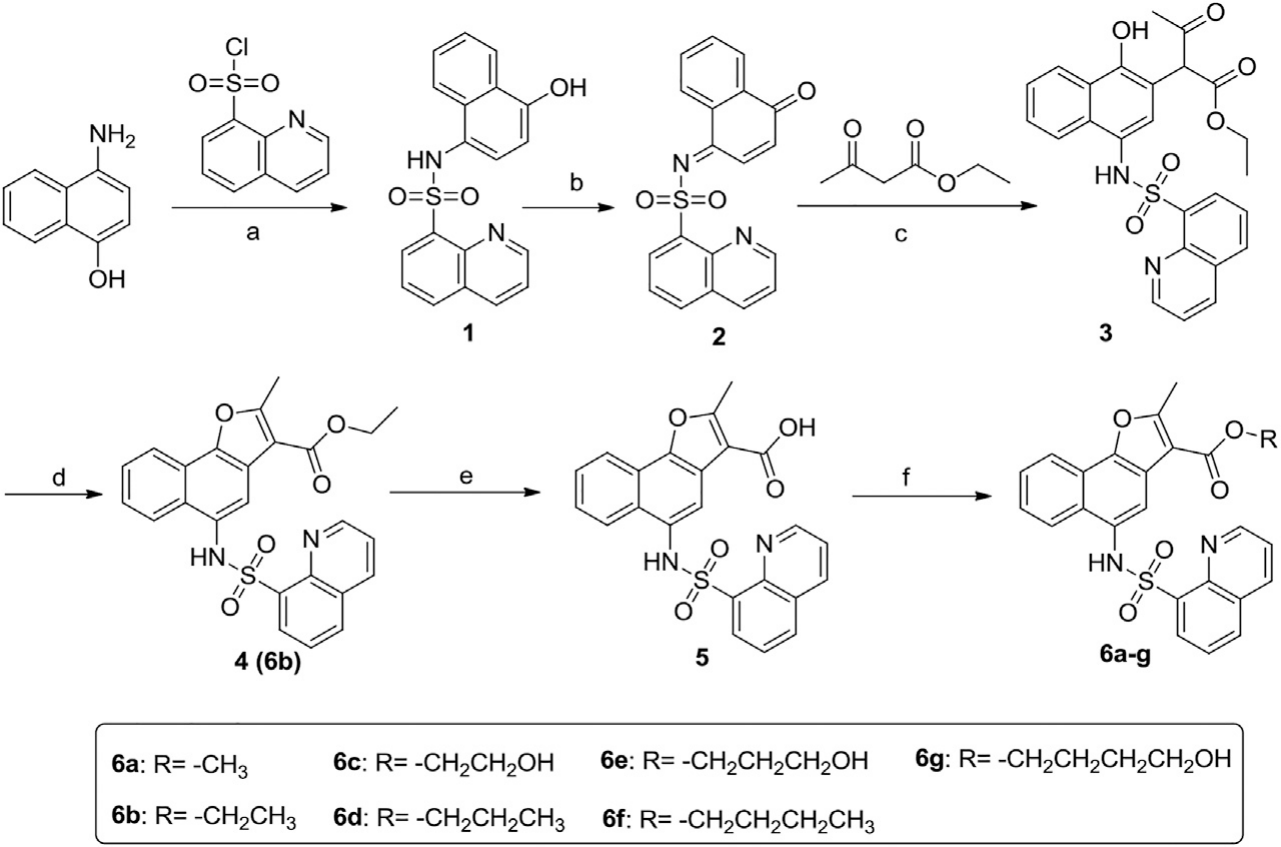
Synthetic route of target compounds 6a-g. Reagents and conditions: **(A)** Pyridine, 0°C; **(B)** K_2_Cr_2_O_7_/acetic acid; **(C)** 1,4-dioxane/CH_3_ONa; **(D)** H_2_SO_4_/CH_3_CH_2_OH; **(E)** 15% K_2_CO_3_, reflux; **(F)** R_1_-OH, H_2_SO_4_.

The synthetic procedure of the target compounds 6a-g was illustrated in Scheme 1. Briefly, 4-amino-1-naphthol was sulfonated with quinoline-8-sulfonyl chloride to prepare intermediate 1, which was then oxidized with K_2_Cr_2_O_7_/acetic acid to give intermediate 2. Further, intermediate 2 was treated with ethyl 3-oxobutanoate to give intermediate 3, which was cyclized under acid catalysis (H_2_SO_4_) to obtain intermediate 4. Intermediate 4 was then hydrolyzed to obtain carboxylic acid intermediate 5, which was subsequently esterified with various alcohols to obtain the target compound 6a-g, respectively.

### Evaluation of Candidate Compounds

**FIGURE 2:**
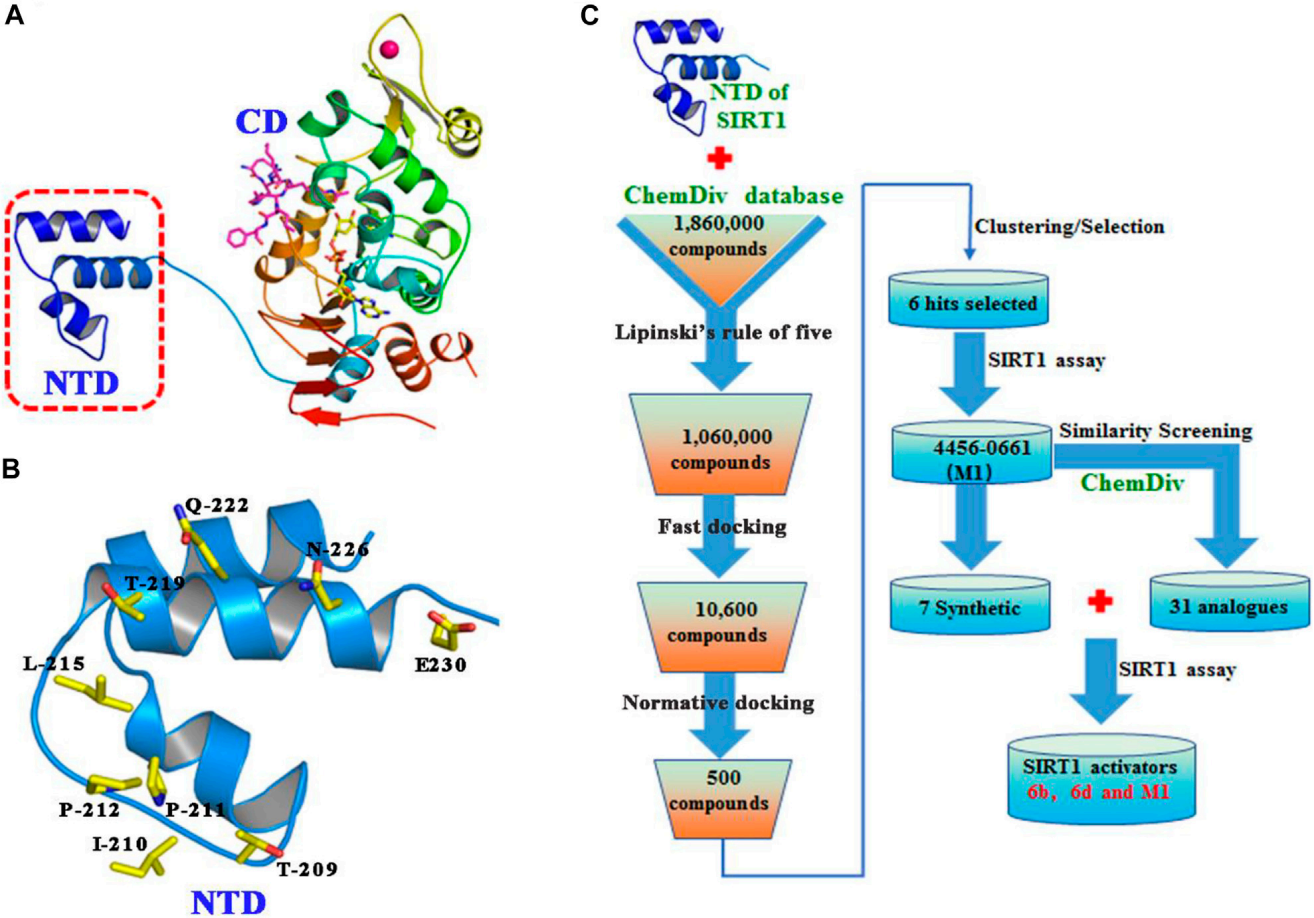
**(A)** Crystal structure of human SIRT1 that is composed of catalytic domain (CD) and N-terminal domain (NTD), and the latter is responsible for the binding of SIRT1 activators. **(B)** Key residues for activator’s binding on the NTD of SIRT1. **(C)** Workflow of the molecular docking-based virtual screening.

The binding pocket formed by Thr209, Ile210, Pro211, Pro212, Leu215, Thr219, Gln222, Asn226, and Glu230 on the NTD of SIRT1 was selected as the potential binding site of SIRT1 activators (Figures 2A,B), and then the ChemDiv chemical library was virtually screened (Figure 2C). Six compounds (Supplementary Figure S1) identified from the VS were tested for their SIRT1 deacetylation activity, with the above-mentioned SRT2104 used as a positive control. Compound M1 showed 215.3% deacetylation activity of SIRT1 at the concentration of 10 μM, which was selected as a potential activator hit for further studies.

### Structure Activity Relationship Analysis for the Analogues of Compound M1

**FIGURE 3:**
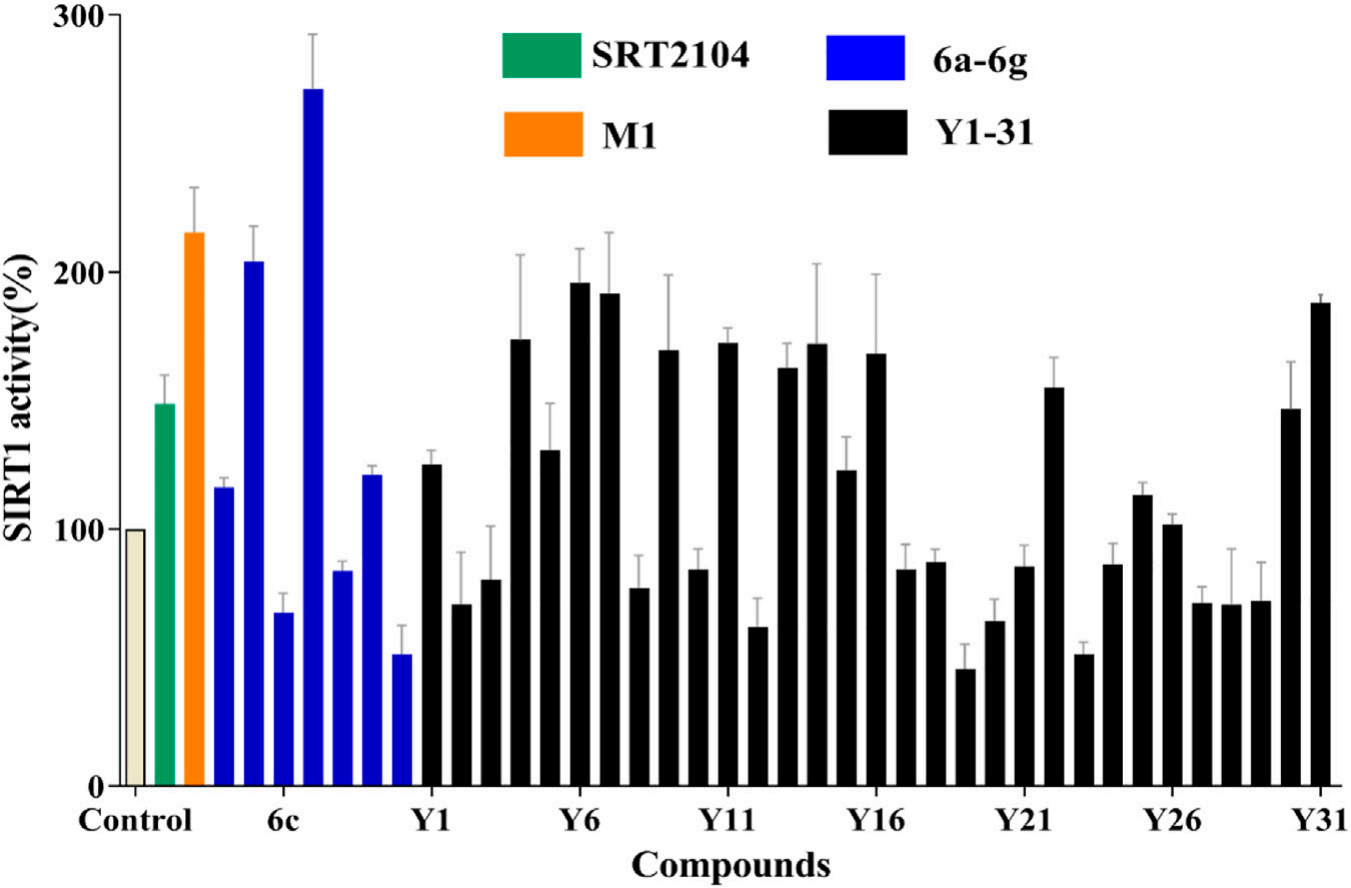
SIRT1 deacetylation activities of lead compound M1 and its analogues Y1-31 and 6a-g at the concentration of 10 μM, with one black control and one positive control SRT2104 (10 μM).

To search for more potent analogues and to explore their initial SAR of the new scaffold of compound M1, similarity-based analogue searching was conducted on the ChemDiv library using compound M1 as the query. A total of 31 analogues (Y1-31) were identified and evaluated by the same activity assessment. The chemical structures of these molecules were shown in Table S1 and their bioactivities were summarized in Figure 3. It is likely that no significant activity improvement was observed, and only compounds Y6, Y7 and Y31 showed comparable bioactivity to compound SRT2104. However, valuable information can be captured from the preliminary SAR analysis, which would provide useful guidance for structural optimization of compound M1.

**FIGURE 4:**
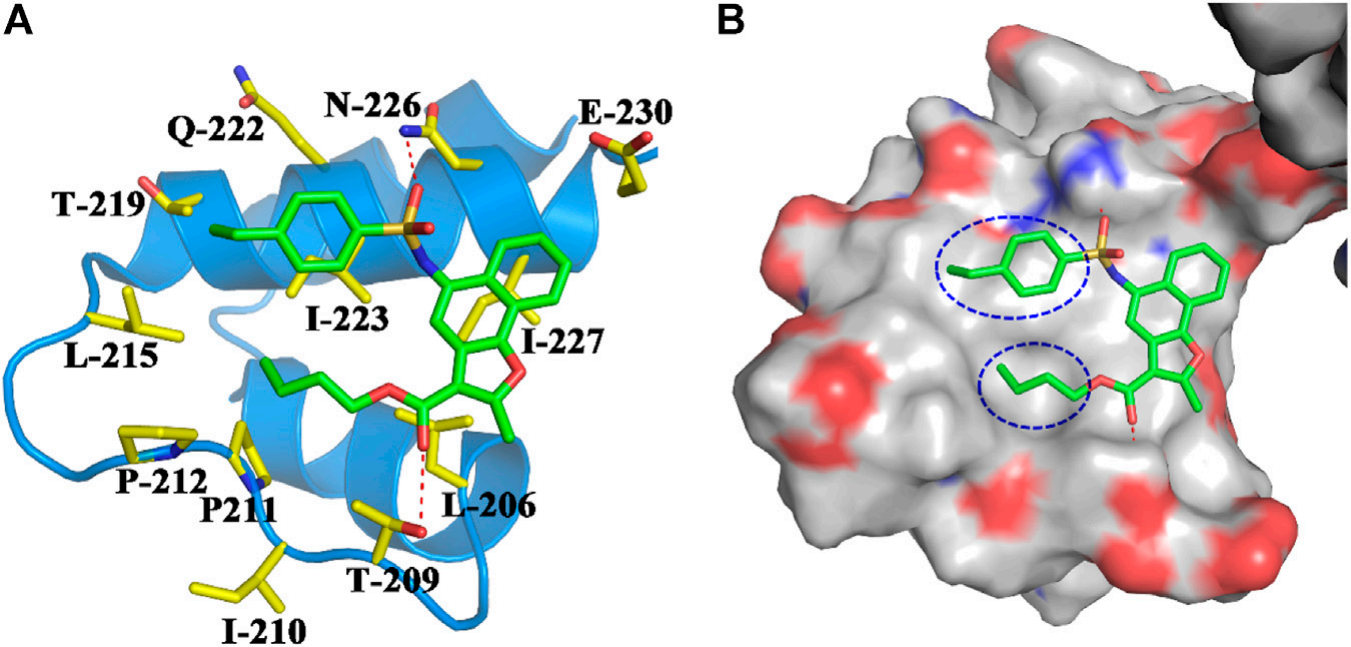
**(A)** Binding mode of compound M1 within the NTD of SIRT1. Compound M1 and the key residues for its binding were all shown in stick model, and colored in green and yellow, respectively. The NTD of SIRT1 was shown in cartoon model and colored in blue. **(B)** Compound M1 bound in the hydrophobic groove of NTD.

According to the predicted binding mode of compound M1 (Figure 4), it formed two hydrogen bonds with the side chains of Thr209 and Asn226 and located exactly in a hydrophobic groove that is composed of the residues Leu206, Ile210, Pro211, Pro212, Leu215, Thr219, Gln222, Ile223, and Ile227. These hydrophobic interactions are most likely the primary driving force for the bindings of compound M1. The bioactivities of the analogues are well consistent with the analysis of the binding modes. For example, the size of the substituents on R_1_ (Figure 1) is critical to ligand binding. Both smaller substituents such as methyl (Y26) and tert-butyl (Y1), and larger substituents such as n-heptyl (Y20–23) and benzyl (Y25), would lead to decreased SIRT1 activity. Not surprisingly, the moderate substituents such as n-butyl (Y30–31) and n-amyl (Y11, Y13–14) had favorable activities. Nevertheless, in view of the fact that the n-butyl group of compound M1 is close to the 4-ethylphenyl group in space, the appropriate size of these two substituents would give rise to more potent SIRT1 activators.

Based on compound M1, we firstly synthesized a series of novel naphthofuran derivatives 6a-g via replacement of the 4-ethylphenyl substituent with quinoline to enhance the hydrophobic interactions with the residues of Thr219, Gln222, and Ile223 in the α3 helix (Figure 4). Simultaneously, the smaller substituents were introduced on R_1_ to avoid creating steric hindrance with the quinoline ring. Excitingly, compound 6d displayed more potent SIRT1 agonistic activity than lead compound M1, while compound 6b was comparable to M1 (Figure 3). Molecular docking predicted binding modes of compounds 6b and 6d (Supplementary Figure S2) were in line with the above structure activity relationship analysis. Compounds 6b and 6d could form an additional hydrogen bond with Asn226 when compared with compound M1. It is noteworthy that compared with compounds 6c, 6e, and 6g, compounds 6b, 6d, and 6f exhibited excellent bioactivities, which could be attributed to the natural hydrophobicity of the binding site as shown in Figure 4B. In other words, the hydroxyl group on the alkyl chain of the ester was unfavorable to insert into the hydrophobic pocket, which resulted in the SIRT1 inhibitory activities for compounds 6c, 6e, and 6g. In addition, SIRT2 and SIRT3 activation assays revealed that the compounds M1, 6b, and 6d were selective activators to SIRT1 (Supplementary Figures S3, S4), which were selected for further bioactivity evaluation.

### *In Vitro* Cell Viability of Compounds M1, 6b, and 6d

**FIGURE 5:**
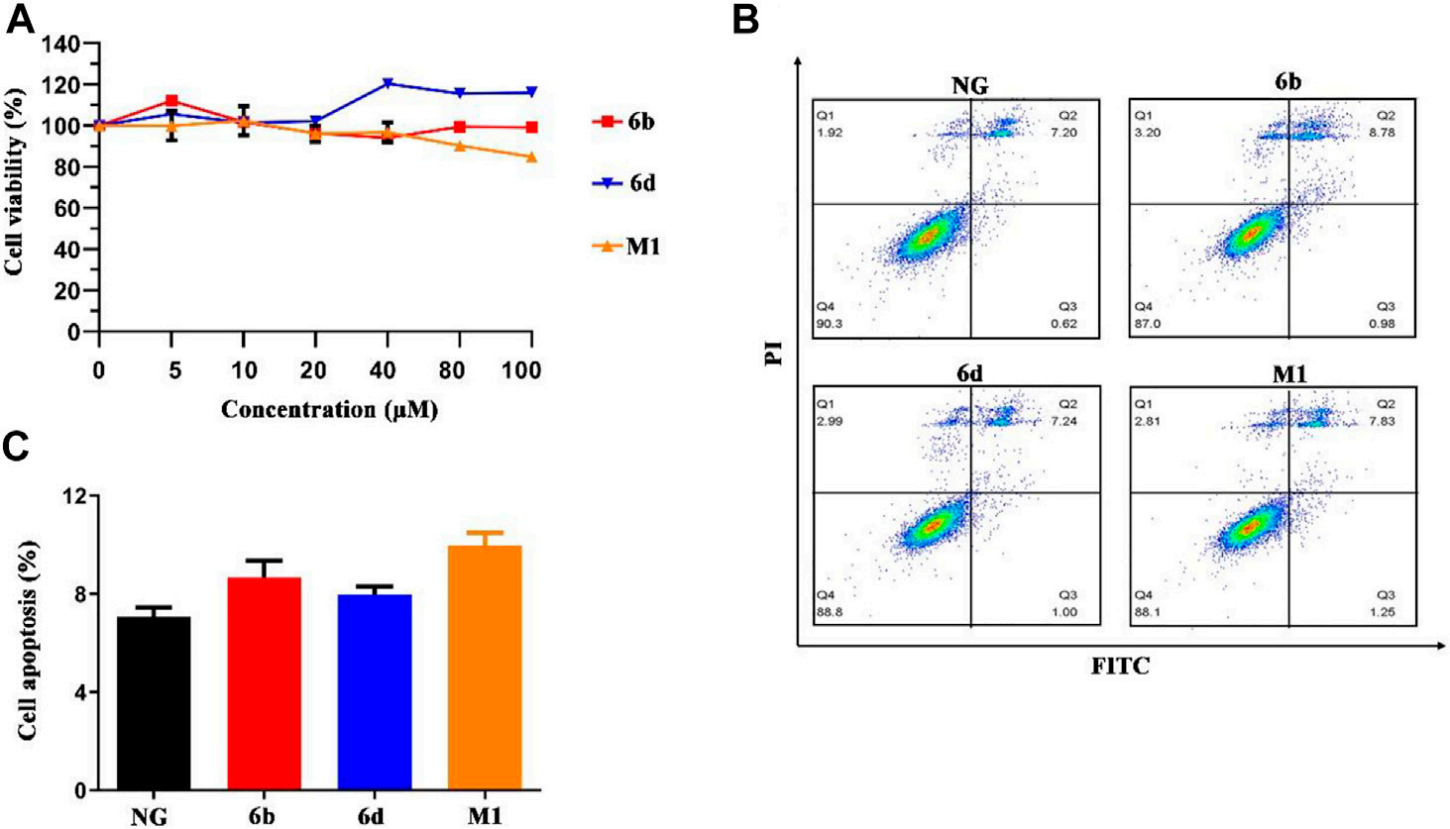
The effect of compounds M1, 6b and 6d on HK-2 cells viability and cell apoptosis. **(A)** Cell viability analysis of HK-2 cells treated with different concentrations of three compounds. **(B, C)** HK-2 cells treated with three compounds (10 μM) were stained with Annexin V-FITC/PI, and flow cytometry analysis showed the ratio of apoptotic cells. The experiments were conducted at least three times. Error bars: mean ± SD.

In order to verify whether these compounds were cytotoxic to normal human kidney cells, human HK-2 and HMC cell lines were selected as target cells, and the compounds were bidirectionally verified by CCK-8 cytotoxicity test and flow cytometry. Briefly, three compounds displayed little or slight intrinsic cytotoxicity to HK-2 and HMC cells (Figure 5A and Supplementary Figure S5A). And our data also showed that the compounds did not induce HMC cell cycle arrest (Supplementary Figure S5B). The results of the flow cytometry assay for apoptosis also showed that compounds M1, 6b and 6d hardly caused apoptosis in HK-2 cells, with the apoptosis rate was below 10% at the concentration of 10 μM (Figures 5B,C). The pro-apoptosis associated proteins were also not activated (Supplementary Figure S5C). Thus, compounds M1, 6b, and 6d possess a relative safety profile. Apart from the *in vitro* cell viability assay, in silico pharmacokinetic prediction using OSIRIS property explorer (http://www.organic-chemistry.org/prog/peo/, accessed on 15 March 2021) were carried out to assess the physicochemical properties of compounds in the cells (Supplementary Table S2). It can be seen that the cLogP values of compounds M1, 6b, and 6d were 5.99, 4.64, and 5.09, respectively, which implied that compound 6b might have an improved absorption and permeation in the cells. The results of solubility and the polar surface area (TPSA) prediction also indicated that compound 6b had enhanced absorption and distribution properties.

### Compounds M1, 6b, and 6d Resist the Apoptosis of HK-2 Cells Induced by High Glucose

**FIGURE 6:**
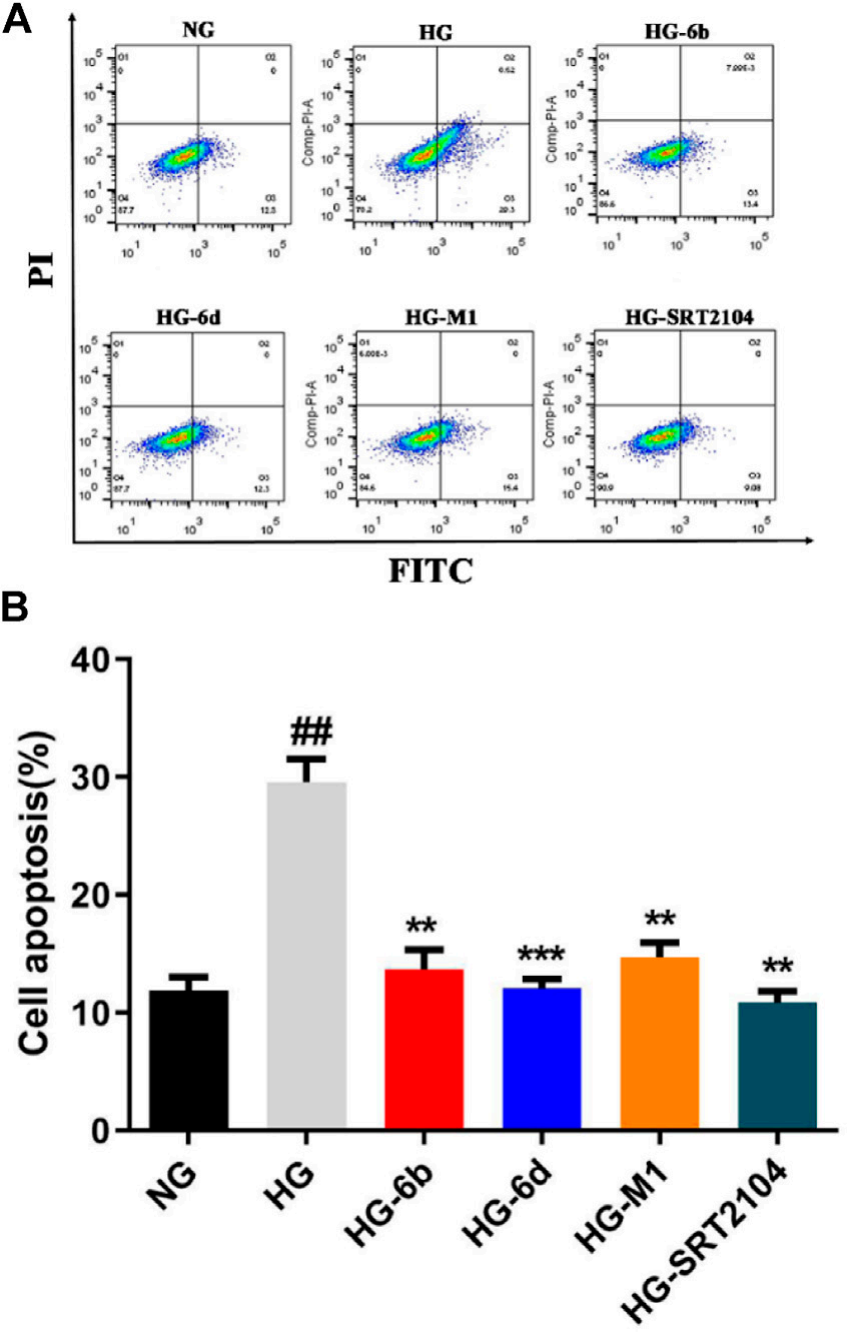
Effect of compounds (10 μM) on HG induced HK-2 cells apoptosis detected by flow cytometry. ^#^High glucose (HG) group compared with Normal glucose (NG) group, *Dosing group compared to HG group, **/^##^ *p* < 0.01, ***p < 0.001.

Previous studies suggested that HK-2 cells’ apoptosis plays an important role in the pathogenesis and progression of DN (Blantz and Singh, 2014), while activation of SIRT1 can significantly deacetylate p53 and reduce HK-2 cells’ apoptosis induced by high glucose (HG) (Brooks and Gu, 2003). To investigate whether these compounds could resist the apoptosis, the flow cytometry, Western blot and real-time quantitative PCR assays were carried out. As shown in Figure 6, compared with the normal glucose (NG) group, the apoptosis of HK-2 cells stimulated by HG was increased obviously, which is consistent with the previous study (Zhou et al., 2015). Impressively, compounds M1, 6b, 6d, and positive drug SRT2104 could significantly decrease the apoptosis caused by HG. Among them, compound 6d had a comparable effect to SRT2104. Surprisingly, at the protein level, high glucose did not activate the canonical apoptosis pathways involving PARP1, Caspase9, BAK, and BAX (Supplementary Figure S6A), which implied that HG induced HK-2 cell death through other pathways.

**FIGURE 7:**
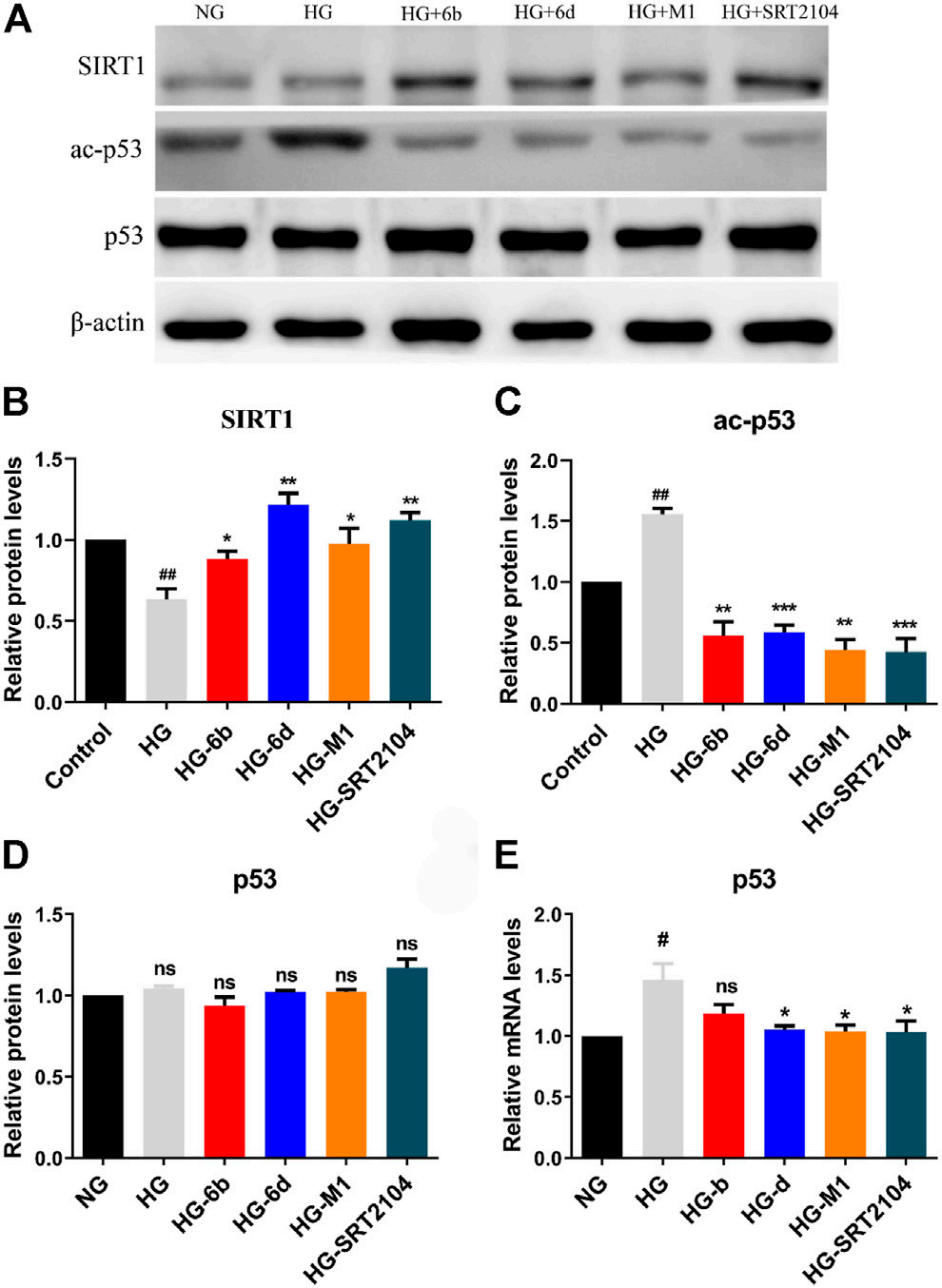
**(A–D)** Western blot analysis protein expression levels of SIRT1, ac-p53, and p53 in HG stimulating HK-2 cells treated with the target compounds (10 μM) for 48 h. **(E)** Q-PCR analysis relative mRNA levels of p53 in HG stimulating HK-2 cells treated with the target compounds (10 μM) for 48 h. ^#^HG compared with NG control group, *Dosing group compared to HG group, */^#^ *p* < 0.05, **/^##^ *p* < 0.01, ***p < 0.001, ns means not significant.

Western blot assay indicated that the SIRT1 protein expression in the HG group significantly decreased when compared with the NG group, whereas the corresponding ones would increase after treatment with compounds M1, 6b, 6d, and positive drug SRT2104 (Figures 7A,B). Moreover, the high expression level of ac-p53 protein inducted by HG was rapidly down-regulated after treatment with the target compounds (Figures 7A,C). Although the expression of p53 protein changed little in all cases (Figures 7A,D), the mRNA levels of p53 in HG-6d, HG-M1, and HG-SRT2104 groups obviously reduced (Figure 7E). Likewise, we found target compounds could reverse the p53 acetylation levels stimulated by HG in HMC cells (Supplementary Figure S6B). Therefore, we speculated that the novel SIRT1 activators M1, 6b, and 6d might inhibit the HG-induce apoptosis of HK-2 cells by activating the SIRT1/p53 pathway.

### Compounds M1, 6b, and 6d Decrease the Inflammation Effect of HK-2 Cells Inducted by HG

**FIGURE 8:**
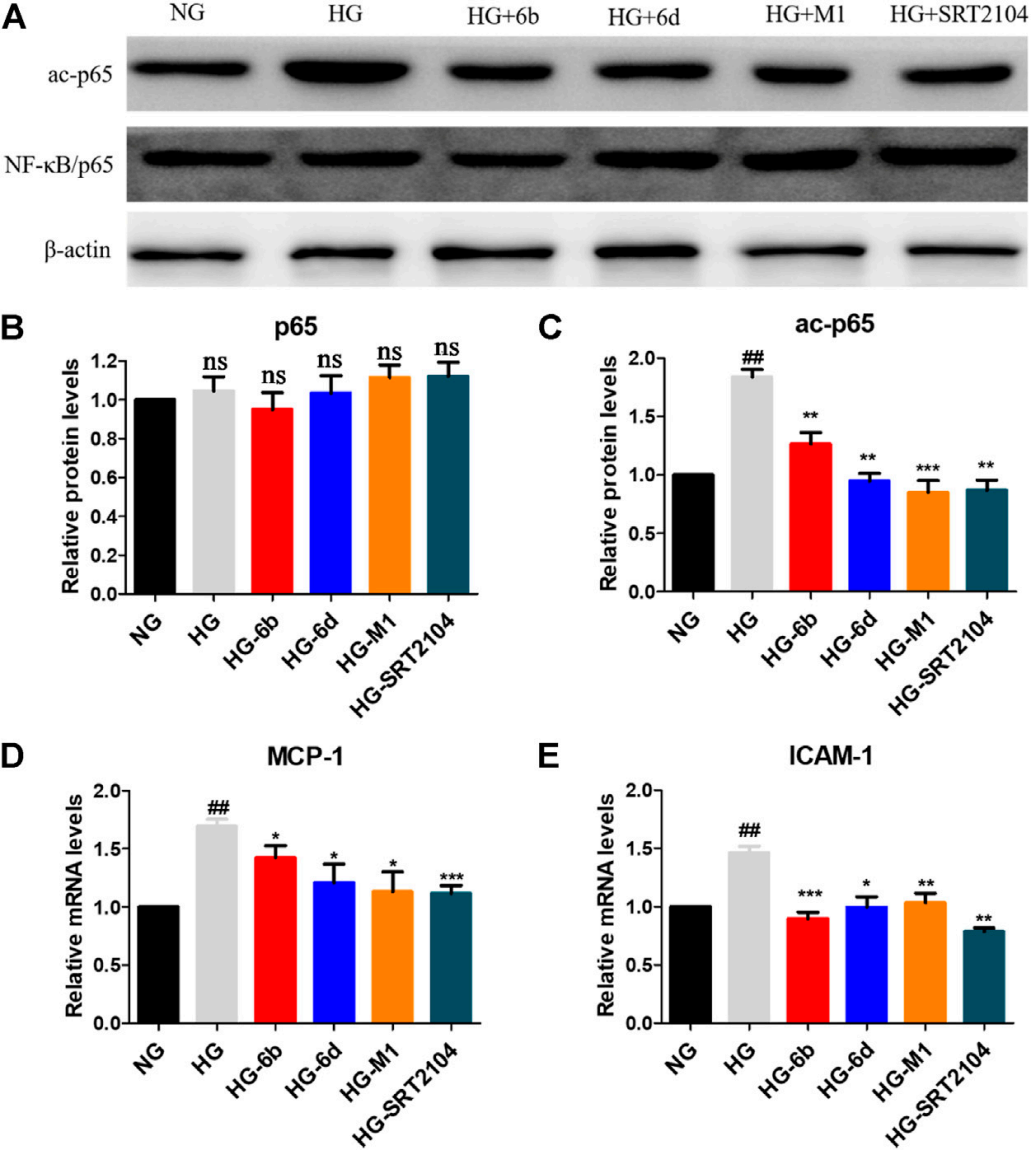
**(A–C)** Effects of the target compounds on protein expression levels of p65 and ac-p65 in HG stimulating HK-2 cells via Western blotting. Cells were treated with the target compounds with dose of 10 μM for 48 h. **(D–E)** Expression of mRNA levels of MCP-1 and ICAM-1 in HG-inducted HK-2 cells when treated with the target molecules (10 μM) for 48 h. ^#^HG compared with NG control group, *Dosing group compared to HG group, */^#^ *p* < 0.05, **/^##^ *p* < 0.01, ***p < 0.001, ns means not significant.

Inflammation is one of the key mechanisms for the occurrence and development of DN (Chung et al., 2006; Navarro-Gonzalez et al., 2011), and nuclear factor kappa B (NF-κB) is an important therapeutic target for preventing kidney damage caused by diabetes (Badal and Danesh, 2014). SIRT1 has been identified as a negative regulator of NF-κB activity by deacetylating Lys310 of NF-κB (p65) (Kiernan et al., 2003). Thus, Western blot and real-time quantitative PCR methods were conducted to evaluate whether the target compounds could relieve the inflammation of HK-2 cells induced by HG. Compared to NG group, HG induction increased the expression of ac-p65 protein after 48 h, while the expression of p65 protein did not vary clearly (Figures 8A–C).

Encouragingly, similar to positive agent SRT2104, compounds M1, 6b and 6d also reduced the protein level of ac-p65 evidently after 48 h (Figures 8A–C). In addition, real-time quantitative PCR assay showed that HG stimulation could promote the expression of downstream of NF-κB, i.e., monocyte chemotactic protein 1 (MCP-1) and intercellular cell adhesion molecule 1 (ICAM-1) in HK-2 cells. While the target compounds could reduce MCP-1 and ICAM-1 mRNA levels effectively. In total, compounds M1, 6b and 6d may alleviate HG-induced inflammation via activating the SIRT1/NF-κB (p65)/MCP-1 pathway.

## Conclusion

Based on the lead compound M1 obtained from virtual screening and bioactivity assay, seven novel SIRT1 activators containing a naphthofuran moiety were designed and synthesized. Most of the target compounds have no toxic effect on HK-2 cells, and compounds 6b and 6d have better cytoprotective effects on HG-induced apoptosis and inflammation of HK-2 cells than lead compound M1 and the positive control SRT2104. Notably, preliminary anti-apoptotic and anti-inflammatory mechanisms indicted that compounds 6b and 6d could not only resist cell apoptosis by activating SIRT1 so as to inhibit the expression of downstream p53 at mRNA and protein levels, but also alleviate cell inflammation by SIRT1/NF-κB/MCP-1 pathway. Taken together, compound M1, 6b, and 6d may be promising candidates in the development of novel SIRT1 activators to prevent apoptosis and inflammation-induced DN.

## Data Availability

The original contributions presented in the study are included in the article/Supplementary Material, further inquiries can be directed to the corresponding authors.
