# Supplementary material for: Design, Synthesis and Pharmacological Evaluation of Naphthofuran Derivatives as Potent SIRT1 Activators
Source: Front Pharmacol. 2021 Apr 28;12:653233. doi: 10.3389/fphar.2021.653233 (PMC8113817; doi:10.3389/fphar.2021.653233)
Supplement: Supplementary file 1 [file DataSheet1.docx]

**Design, synthesis and pharmacological evaluation of naphthofuran derivatives as potent SIRT1 activators**

Jian Gao^a,b^, Qingqing Chen^a^, Ye Huang^a^, Kaihang Li^a^, Xiaoju Geng^a^, Tao Wang^e^, Qisi Lin^a,*^ and Ruosi Yao^c,d,^*

*^a^Jiangsu Key Laboratory of New Drug Research and Clinical Pharmacy,* *Xuzhou Medical University, Xuzhou, Jiangsu 221004, P. R. China*

*^b^Xuzhou Medical University Technology Transfer Center Co., Ltd., Xuzhou Medical University, Xuzhou, Jiangsu 221004, P. R. China*

*^c^Department of Hematology, The Affliated Hospital of Xuzhou Medical University, Xuzhou, Jiangsu, China*

*^d^ Blood Diseases Institute, Xuzhou Medical University, Xuzhou, Jiangsu, China*

*^e^Jiangsu College of Nursing, Huaian, Jiangsu 223005, P. R. China*

*Corresponding author: Ruosi Yao

E-mail: yaors908@163.com

Tel.: +86 0516 85802382

Fax: + 86 0516 85802382

*Corresponding author: Qisi Lin

E-mail: qslin074@126.com

Tel.: +86 051683262137

Fax: + 86 0516 83262137

**Figure S1.** Chemical structures and SIRT1 deacetylation activities (10 μM ) of six candidates obtained from molecular docking-based virtual screening. ChemDiv database (commercially available and the purity of compounds are greater than 95%) of TopScience Co., Ltd. (Shanghai, China) was selected as a screening library.

**Table S1** Chemical structures of compound 4456-0661 (M1) derivatives obtained from the similarity-based virtual screening conducted on the ChemDiv database.

| No. | R_2_ | R_1_ |
| --- | --- | --- |
| Y1 | 4-ethylphenyl | -CH(CH_3_)_2_ |
| Y2 | 4-(tert-butyl) phenyl | -CH_2_CH_2_OCH_3_ |
| Y3 | 2,4-dimethylphenyl | -CH_2_CH_2_OCH_3_ |
| Y4 | 2,4,6-trimethylphenyl | -CH_2_CH_2_OCH_3_ |
| Y5 | 4-bromophenyl | -CH_2_CH_2_OCH_3_ |
| Y6 | 4-ethylphenyl | -CH_2_CH_2_OCH_3_ |
| Y7 | 2,5-dimethylphenyl | -CH_2_CH_2_OCH_3_ |
| Y8 | 4-methylphenyl | -CH_2_CH_2_OCH_3_ |
| Y9 | phenyl | -(CH_2_)_7_CH_3_ |
| Y10 | 4-methoxyphenyl | -(CH_2_)_4_CH_3_ |
| Y11 | 4-ethoxyphenyl | -(CH_2_)_4_CH_3_ |
| Y12 | 2,4-dimethylphenyl | -(CH_2_)_4_CH_3_ |
| Y13 | 4-fluorophenyl | -(CH_2_)_4_CH_3_ |
| Y14 | 4-bromophenyl | -(CH_2_)_4_CH_3_ |
| Y15 | 2,5-dimethylphenyl | -(CH_2_)_4_CH_3_ |
| Y16 | naphthalene | -(CH_2_)_4_CH_3_ |
| Y17 | 4-fluorophenyl | -(CH_2_)_3_CH_3_ |
| Y18 | 2,5-dimethylphenyl | -(CH_2_)_3_CH_3_ |
| Y19 | 4-methoxyphenyl | -(CH_2_)_3_CH_3_ |
| Y20 | 4-methylphenyl | -(CH_2_)_6_CH_3_ |
| Y21 | 4-ethylphenyl | -(CH_2_)_6_CH_3_ |
| Y22 | 4-methoxypheny | -(CH_2_)_6_CH_3_ |
| Y23 | phenyl | -(CH_2_)_6_CH_3_ |
| Y24 | 4-ethoxyphenyl | -(CH_2_)_2_CH_3_ |
| Y25 | 4-methylphenyl | -CH_2_C_6_H_5_ |
| Y26 | naphthalene | -CH_3_ |
| Y27 | 2,4,5-trimethylphenyl | -CH_2_CH_2_OCH_3_ |
| Y28 | 2,4,5-trimethylpheny | -(CH_2_)_4_CH_3_ |
| Y29 | 2,4,5-trimethylphenyl | -(CH_2_)_3_CH_3_ |
| Y30 | thiophene | -(CH_2_)_3_CH_3_ |
| Y31 | 2,5-dimethoxyphenyl | -(CH_2_)_3_CH_3_ |

**Table S2** In silico pharmacokinetic prediction study of compounds M1, 6b, and 6d.

| Parameters | M1 | 6b | 6d |
| --- | --- | --- | --- |
| Solubility^a^ | -7.57 | -7.23 | -7.5 |
| CLogP^b^ | 5.99 | 4.64 | 5.09 |
| Molecular weight | 465.0 | 460.0 | 474.0 |
| TPSA^c^ | 93.99 | 106.8 | 106.8 |

^a^ Solubility: estimated logS value, a unit stripped logarithm (base 10) of the solubility measured in mol/L; ^b^ cLogP: logarithm of the partition coefficient of the compound between n-octanol and water log (octanol/water); ^c^ TPSA: the polar surface area prediction is based on an atom-type based increment system, published by P. Ertl, B. Rohde, P. Selzer in J. Med. Chem. 2000, 43, 3714-3717.


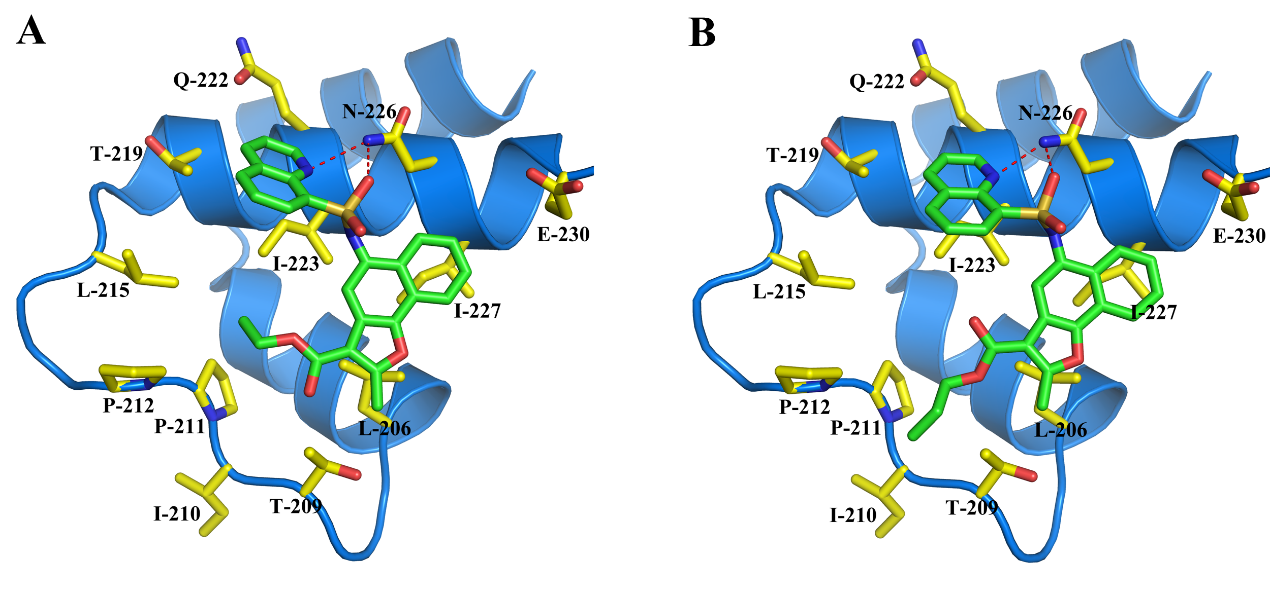


**Figure S2.** The predicted binding modes of compounds 6b (A) and 6d to SIRT1. The protein SIRT1 was shown in cartoon model and colored in blue. The target compounds and the key residues for their binding were shown in stick and colored in green and yellow, respectively.


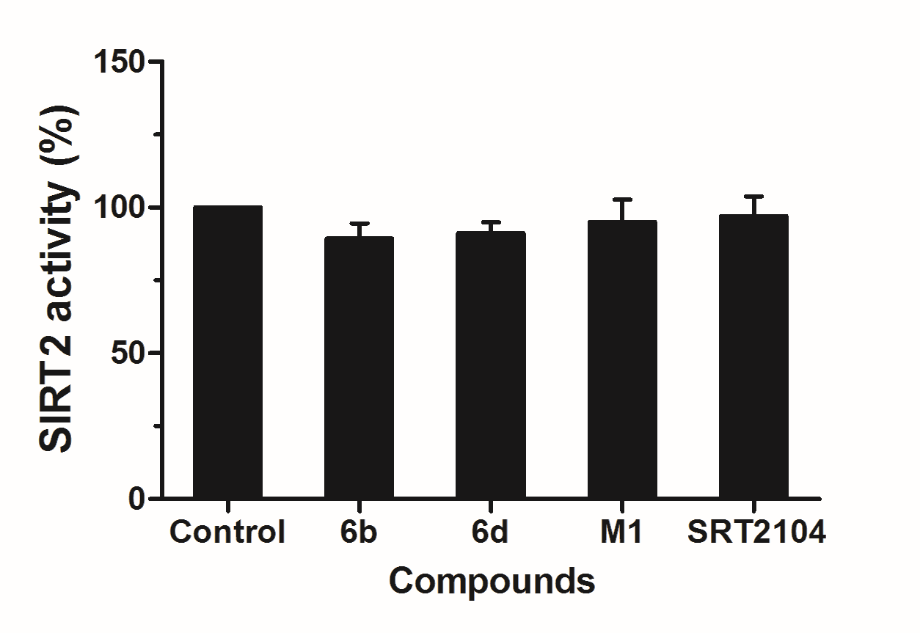


**Figure S3.** SIRT2 deacetylation activities of compounds M1, 6b and 6d at the concentration of 10 μM, with one black control and one positive control SRT2104 (10 μM).


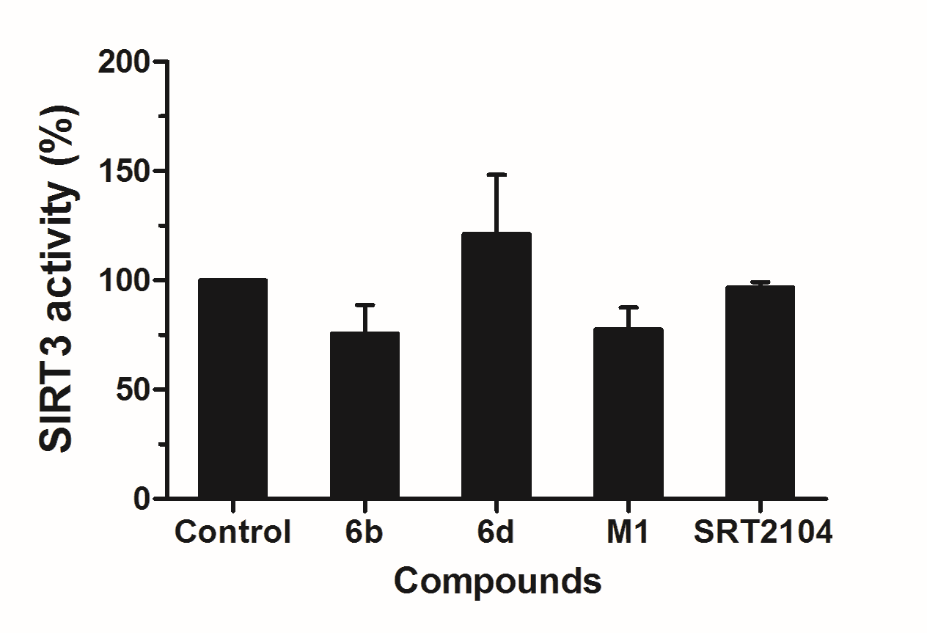


**Figure S4.** SIRT3 deacetylation activities of compounds M1, 6b and 6d at the concentration of 10 μM, with one black control and one positive control SRT2104 (10 μM).


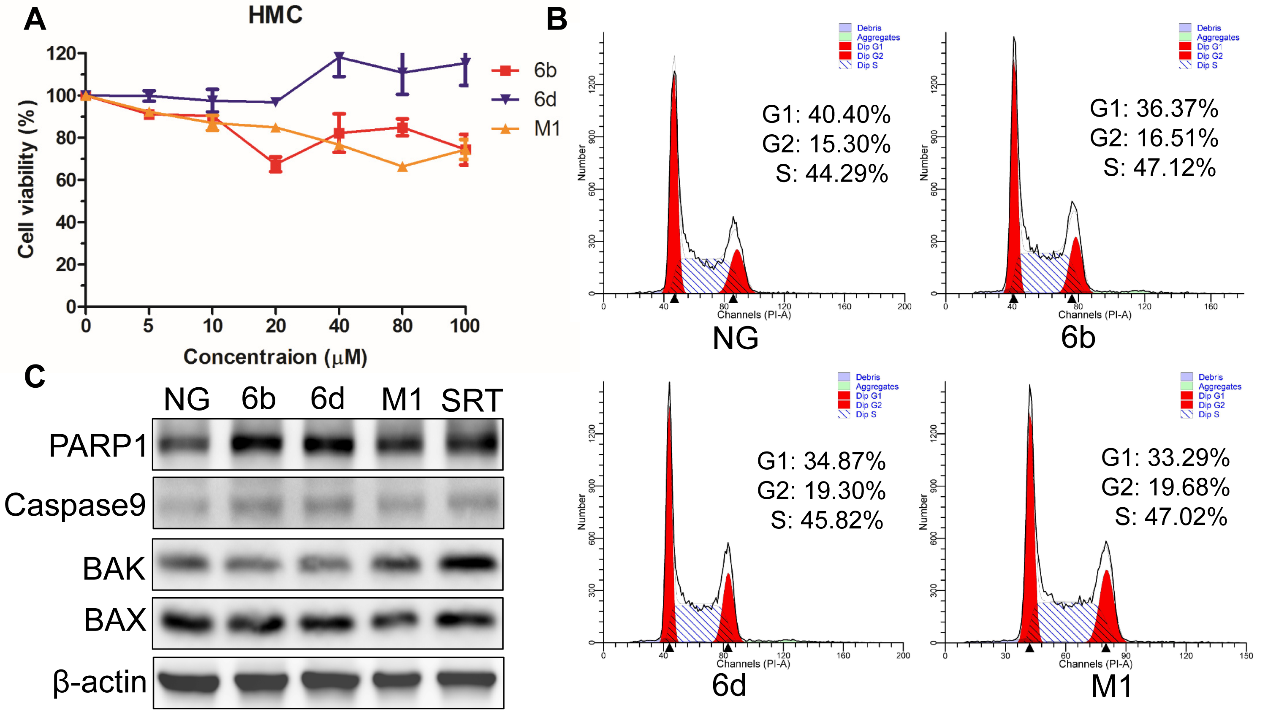


**Figure S5.** The effect of compounds on HMC cell viability and cell cycle. (A) Cell viability analysis of HMC cells treated with different concentrations of three compounds. (B) HMC cells treated with three compounds (10 μM) were stained with PI, and flow cytometry analysis showed HMC cell cycle distribution. (C) Western Blot analysis of apoptosis-related proteins, with β-actin used as an internal control. SRT: SRT2104, as positive drug.


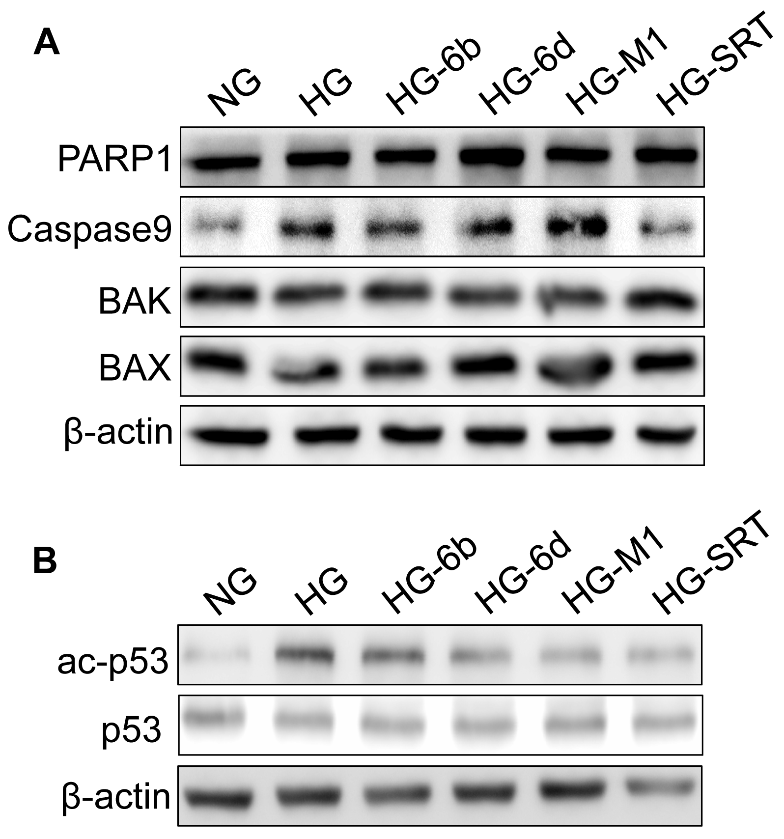


**Figure S6.** The effect of compounds on apoptosis-related proteins. (A) Western Blot analysis of the expression level of PARP1, Caspase9, BAK and BAX in HK-2 cells treated by HG and compounds. β-actin was used as internal controls. (B) Western Blot analysis of p53 and corresponding acetylated p53 in HMC cells treated by HG and compounds. β-actin was used as internal controls.
